# Screening of Piglets for Signs of Inflammation and Necrosis as Early Life Indicators of Animal Health and Welfare Hazards

**DOI:** 10.3390/ani15030378

**Published:** 2025-01-28

**Authors:** Karien Koenders-van Gog, Thomas Wijnands, Mirjam Lechner, Gerald Reiner, Johanna Fink-Gremmels

**Affiliations:** 1 Lintjeshof Veterinary Practice, LH Vet Group, 6031 RK Nederwert, The Netherlands; k.koenders@lintjeshof.com (K.K.-v.G.); t.wijnands@lintjeshof.com (T.W.); 2 UEG Hohenlohe, 91567 Herrieden, Germany; mirjam.lechner@web.de; 3 Clinic for Swine—Herd Health Management and Molecular Diagnostics, Justus-Liebig-University Giessen, 35392 Giessen, Germany; 4 IRAS—Department Population Sciences, Faculty of Veterinary Medicine, Utrecht University, Yalelaan 104, 3584 CM Utrecht, The Netherlands; j.fink@uu.nl

**Keywords:** pigs, inflammation, necrosis, animal health, animal welfare, diagnostics

## Abstract

Ensuring animal health and well-being requires animal-based measures for early and direct intervention at the point of care. Insight into the pathophysiology of Swine Inflammation and Necrosis Syndrome (SINS) has led to a non-invasive, clinical scoring system that can be used in daily practice. This provides information on the cause of observed lesions for direct intervention in affected herds. The aim of the current study was to re-evaluate the practicality of the SINS scoring system under field conditions. In addition, this is the first study to provide insight into the prevalence of SINS on Dutch farms. This study involved the scoring of 5958 piglets from 20 visits on 13 farms randomly selected by a veterinary practice in the Netherlands. The results showed that up to 64.1% of the piglets had visible inflammatory alterations in different body parts within the first seven days of life. Sow rectal temperature, signs of coprostasis and water intake during pregnancy were significantly (*p* ≤ 0.05) correlated with the prevalence of SINS in piglets. In conclusion, the SINS scoring system is an easy-to-use, non-invasive diagnostic tool that summarises animal-based observations at the point of care, providing a valuable communication tool between farmers, nutritionists and veterinarians in their efforts to improve animal health and welfare.

## Simple Summary

As an animal-based measure, signs of inflammation and necrosis in pigs (SINS) can be recorded easily, early in life and non-invasively. The present study confirms the good diagnostic applicability of SINS in practice, provides a first overview of the occurrence of the syndrome on Dutch farms and indicates underlying causes that can guide farmers, nutritionists and veterinarians to implement strategies to improve animal health and welfare.

## 1. Introduction

Caring for animal health and welfare is an ethical and societal requirement delegated to all professionals involved in the daily care and supervision of farm animals [1]. Animal behaviour, welfare and health are determined by a combination of inherited traits (natural behaviour) and the given husbandry conditions on a farm, as assessed by resource-based measures (RBMs [2]). Modulated by physiological factors such as breed, sex and age, these input parameters determine the response of the animal [3,4]. In turn, animal care must combine the assessment of RBMs with direct clinical observation of the animals themselves (Animal-based measures, ABMs [5]), as only through such an integrated approach can animal welfare be truly and reliably assessed. This combined approach at the point of care, i.e., directly on the farm, will improve our understanding of the impact of housing conditions and farm management on the overall health, welfare and productivity of farm animals and allow corrective action to be taken where necessary. For example, if animals are suffering from laminitis (assessed using ABMs), it is possible that this is simply a response to poor flooring conditions (an RBM) [6,7]. However, clinically observed lameness and claw or heel lesions in piglets may also be indicative of a generalised inflammatory response resulting from early life dysbacteriosis or stress-induced leaky gut syndrome [8]. The pathogenesis of such generalised inflammatory responses involves the impairment of intestinal barrier integrity and subsequent translocation of bacterial toxins and even bacteria from the intestinal lumen into the portal circulation and beyond [9,10,11,12,13]. Leaky gut syndrome in early life may have multiple causes, reflecting the vulnerability of the gut to hypoxia at birth, the adaptation of the gut to feed utilisation, accompanied by the initial colonisation of the gut by microbes and the establishment of an intestinal microbiome [14]. In addition, feed-borne toxins, including mycotoxins, some of which are also excreted in sow milk [15,16], and multiple viral and bacterial infections in early life may contribute to this inflammatory response [17,18,19,20,21].

The disruption of intestinal barrier function and mild endotoxemia are well recognised risk factors for the development of a disease in pigs described as Swine Inflammation and Necrosis Syndrome (SINS) [22].

Based on the clinical, thermographic [23] and pathohistological [8,24] identification of inflammatory processes in piglets, the SINS scoring system is applied in newborns, suckling piglets, in the postweaning phase and even in later life stages as an animal-based health and welfare indicator [24]. Furthermore, the SINS scoring system is considered to be one of the ABMs that can predict the risk of tail biting, one of the most common and devastating behavioural disorders in modern pig production, as the molecular events recognised in SINS have also been shown to trigger sickness behaviour and tail biting [25].

SINS scoring of very young piglets (2–7 days old) is non-invasive and easy to perform and implement by trained personnel in daily practice, as piglets are routinely handled at this age and because the signs can be obtained by clinical observation alone, without the need for interventions, elaborate sampling or equipment. The uncertainty associated with subjectivity when using a clinical/visual scoring system could be limited by the creation of an international standard (with comparative training sessions). Moreover, the SINS score is ultimately composed of multiple observations that can be clearly distinguished as present or absent (e.g., redness, swelling, bleeding, etc.) recorded as binary data. However, minor differences between different studies may arise from modification in the scoring matrix. For this reason, we included a picture gallery of the clinical findings in this manuscript and added some references to these parameters [8,26,27,28]. In addition, SINS scoring at this early age allows diagnostic differentiation between clinical signs obviously related to sow management, when these clinical signs are observed as early as their first day of life [8], and environmental factors such as hygiene, temperature and water management in the farrowing unit. Scoring piglets at the point of care (i.e., at the individual farm and group level) allows for early and targeted intervention. Scoring piglets at the point of care (i.e., at the individual farm and group level) was used as a diagnostic tool to allows for an early and targeted intervention.

While the behavioural changes that occur later in life, such as overt aggressiveness and tail biting, have been well described [29], the SINS protocol includes the observation of several other body sites that may be affected by welfare-impairing inflammatory lesions [22]. These additional body parts include the ears, teats, umbilicus and coronary bands, which are added to the heel and claw scores to serve as welfare indicators and help identify potential causes of SINS and consecutively select corrective measures.

The simultaneous occurrence in such different parts of the body and the variable signs of inflammation, which can progress to necrosis, led to the definition of SINS [26]. The inflammatory nature of the syndrome was pathohistologically confirmed in two studies, one in suckling piglets, weaners and finishers [24] and a second in newborn piglets [8]. As a clear sign of inflammation, the invasion of macrophages and lymphocytes into clinically visible sites of inflammation was observed in newborn piglets as early as two hours after birth. It was concluded that the initiation of this inflammatory syndrome must have occurred at least several days before birth [30]. These observations were confirmed by studies on the association of SINS with clinical–chemical and metabolic parameters [31] and by profiling of the metabolome and liver transcriptome [32,33]. A further indication of the primarily endogenous nature of the syndrome was the demonstration of a pronounced heritability, including symptoms in the claw area [27].

The high prevalence of SINS in commercial pig nurseries and farms is noted and discussed in several of these studies. For example, in the study by Kühling et al. [8], 85% of the piglets examined had visible lesions on clinical examination in at least five of the nine body parts scored. Changes ranged from redness to swelling, inflammatory bristle, exudation and necrosis and were observed in piglets, weaners and even finishers, although the prevalence often varied considerably. Changes at the base of the tail were found in 48.7%, 31.9% and 4.9% of newborn piglets, weaners and finishers, respectively. The tail tip was affected in 32.2, 68.1 and 21.4%, the ears in 63.5, 76.1 and 31.1%, the teats in 75.7, 56.6 and 1% and the coronary band in 94.8, 13.3 and 0% of the corresponding age groups [24]. A study from France (n = 2377 piglets from 16 herds) described a lesion prevalence of 85.8% in piglets. Individual scores indicated heel lesions in 61.4%, coronary band lesions in 58.6%, tail lesions in 23.5%, head skin oedema in 16.9%, teat lesions in 12.9% and ear lesions in 10.8% of 2–3-day-old suckling piglets [28]. These results are consistent with initial results from Germany [26]. In another study conducted by a breeding company, including approximately 5700 piglets aged 2–3 days from a German farm, inflammation and necrosis of the ears, tail, teats and claws were found in 19%, 16.9%, 14% and 43.5% of the animals, respectively, resulting in a total of 62% SINS-positive piglets in this cohort [27].

In light of these previous findings, the aim of the current study was to re-evaluate the practicability of the SINS scoring system under field conditions in the Netherlands and to gain additional insight into the prevalence of SINS on Dutch farms.

## 2. Materials and Methods

Clinical signs of SINS were scored in 5985 piglets during 20 visits to 13 Dutch pig herds randomly selected by a local veterinarian. This study was conducted between July 2022 and January 2024. Four sow and seven boar genotypes were used in investigated herds in a total of 14 combinations. The sow genotypes were TN70 (Topigs Norsvin, Den Bosch, The Netherlands), Hypor (Boxmeer, The Netherlands), Deen (Danbred, Vejle, Denmark), TN York (Topigs Norsvin). The most frequently used boars were TEMPO (Topigs Norsvin), Maxter (Hypor), Duroc (Danbred), Norsvin Landrace (Topigs Norsvin). Due to the multiple combination and the non-resolvable interaction between farm management and genetics, the genetics could not be considered in the statistical evaluation. Only controlled studies, with defined herds and multiple rounds of piglets in individual farms, could clearly define the influence of genetical and environmental factors on the outcome of the SINS scoring.

On all farms, piglets in one or more farrowing batches were clinically examined during their first week of life (mean age 3.3 days). The piglets’ tails were undocked at the time of assessment. All clinical scoring was performed by the same two individuals at the same time to minimise bias.

**Figure 1:**
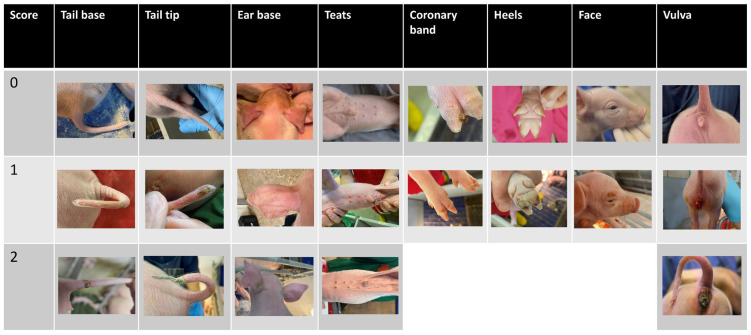
Examples for the SINS scores as applied in the present study.

The prevalence of inflammation and necrotic lesions on the tail tip and base, ear base, claw coronary bands, heels, vulva, face and teats was determined by clinical assessment using a predefined qualitative scoring matrix (Table 1 and Figure 1), adapted from Reiner et al. [22].

The scores were calculated at individual piglet and herd levels. To calculate the total score for each piglet, the sum of the lesions was calculated and divided by the maximum possible score, 15 for female piglets and 13 for male piglets (excluding the 2 points for positive vulvar lesions) according to Table 1.

It was not possible to include husbandry and feeding parameters because they were present in too many varieties on only a few farms. On a subset of 13 farm visits, additional parameters were recorded, including sow rectal temperature at the time of scoring, presence or absence of coprostasis, average water intake per day and sow during pregnancy, uterine discharge and piglet losses until weaning.

Statistics were calculated with IBM SPSS, Version 27. Prevalence was calculated as the proportion of affected piglets in each case. SINS scores were calculated in two ways: firstly, including lower leg lesions (SINS+LL; including coronary bands and heels) and, secondly, excluding these lower leg lesions (SINS−LL). The rationale for this approach was to allow the influence of the flooring conditions to be examined and quantified over time. The prevalence of SINS signs was categorised by visit. This was performed to describe herd effects at the respective visit. The cumulative development of SINS prevalence was shown by adding the affected animals in the affected herds, separately for SINS+LL (grey line) and SINS−LL (black line). The significance of binary data (e.g., presence or absence of redness) was tested using the Chi^2^ test. Body part scores and SINS scores were normally distributed. Effects on their distribution were analysed using a generalised linear model. The effects of herd, day of life, parity and sex of the piglets were included in the model. In addition, sow number was included as a random effect and piglet birth weight as a covariate. Correlations were calculated as Spearman correlations. Finally, the 20th percentile of herds with the lowest and highest SINS scores were selected to examine the sow traits of coprostasis and uterine discharge by Chi^2^ test and the sow traits of body temperature, water intake, parity and preweaning losses in these groups by one-way analysis of variance. Effects with a *p* value ≤ 0.05 were treated as statistically significant.

## 3. Results

### 3.1. Prevalence of SINS

**Figure 2:**
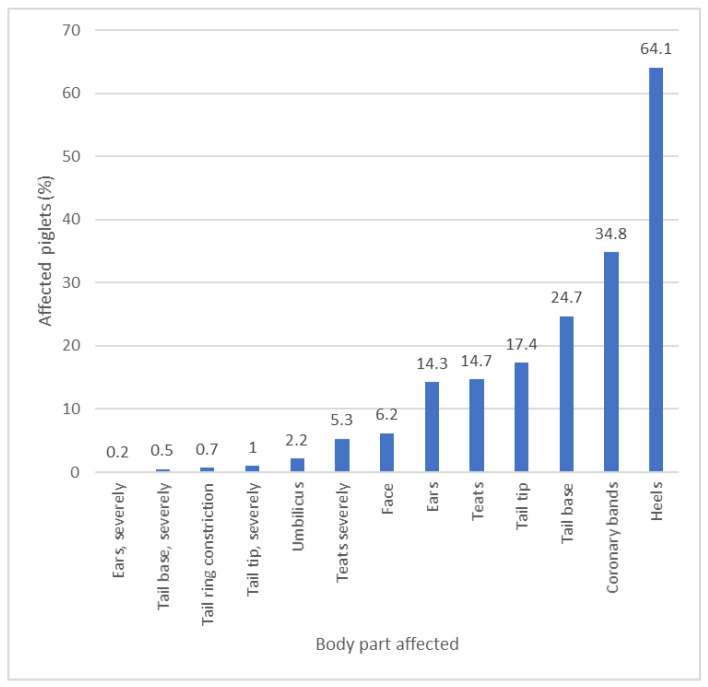
Prevalence of SINS signs on predisposed body parts of 5958 piglets scored in the first week of life (1–7 days postpartum) on 13 individual farms.

Complete SINS scoring data were available for 5958 suckling piglets from 13 different farms. Signs of SINS in at least one part of the body were recorded in 83.7 ± 17.1% of all piglets. The highest number of lesions was found in the heels, with an average of 64.1% of all piglets affected (Figure 2). The coronary band was affected in 34% of the piglets. The prevalence of SINS in other parts of the body ranged from 0.2% to 24.7%. In view of the very high values for lesions on the lower legs, a targeted analysis of the data was carried out to determine the possible influence of the housing conditions and flooring in the farrowing units on the overall percentage of SINS-positive piglets.

### 3.2. Distribution of Clinical Signs of SINS on Individual Farms Potentially Related to Housing Conditions

The prevalence of clinical signs of SINS on individual farms is summarised in Table 2. In addition to the individual data, the last two columns present two distinct subgroups of SINS-positive piglets, designated as SINS+LL, reflecting the total percentage of piglets including those with lower leg lesions at the heels and coronary bands, and SINS−LL as the percentage of piglets without lower leg lesions.

**Figure 3:**
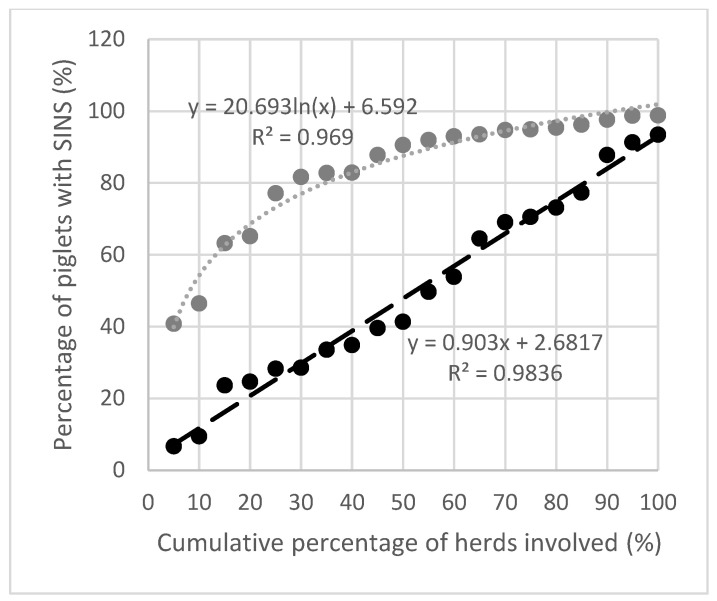
The cumulative development of SINS prevalence is shown by adding the affected animals in the affected herds, separately for SINS+LL (grey line) and SINS−LL (black line). When coronary bands and heels (LL) were included, 46.5% of piglets were affected in the top 10% of herds and 80% of piglets were affected in the top 30% of herds. Excluding LL, only 10% of piglets in the top 10% of herds were affected.

The prevalence of piglets with SINS+LL followed a logarithmic distribution (Figure 3). There was a linear relationship for SINS−LL with 40% of piglets affected on at least 50% of the farms. Both correlations had a high coefficient of determination (0.969 for SINS+LL; 0.984 for SINS−LL).

### 3.3. Distribution of Clinical Signs of SINS Attributable to Systemic Inflammation on Individual Farms

While lesions on the heels and coronary bands (lower legs) may be caused by environmental conditions (RBMs), such as inappropriate flooring in the farrowing unit, all other lesions routinely scored in the SINS matrix refer to inflammatory lesions at typical sites of predilection for inflammation, but without a direct link to the piglet’s environment.

Piglets from 20% of herds with the highest SINS scores were affected at their tail base in 67% of cases (Table 3). The corresponding values for piglets from 20% of herds with the lowest SINS scores were only 1–3%.

### 3.4. Age Dependency of Clinical Signs of SINS in Piglets

**Figure 4:**
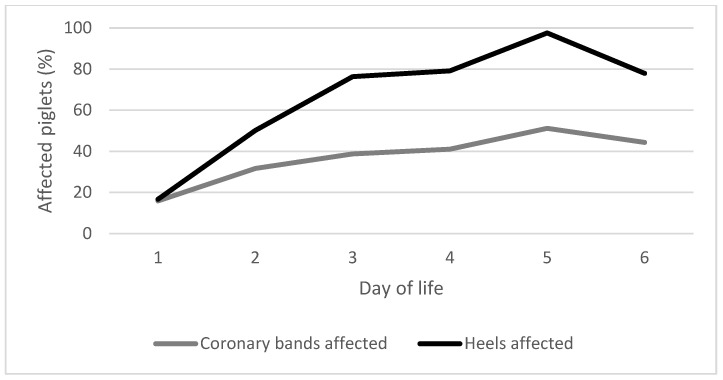
Increase in the prevalence of inflammation/necrotic lesions in individual farms and coronary bands and heels), during the first 6 days of life.

SINS+LL and SINS−LL scores in 1 to 7-day-old piglets showed a highly significant correlation (r = 0.815; *p* < 0.0001) with the actual day of scoring and therefore with the age of the piglet. This level of correlation remained stable over time (Table 4). However, it should be noted that non-floor-associated sites (SINS−LL) did not show any significant changes during the first 7 days of life, whereas the prevalence of inflammation of floor-associated sites (SINS+LL) increased significantly during this period (Figure 4). Correlations between day of life and inflammation of the heels (r = 0.588; *p* < 0.001) and coronary arteries (r = 0.257; *p* = 0.024) were significant. For all other parts of the body, there was no directional relationship with day of life.

### 3.5. Gender Distribution of Clinical Signs of SINS in Piglets

**Figure 5:**
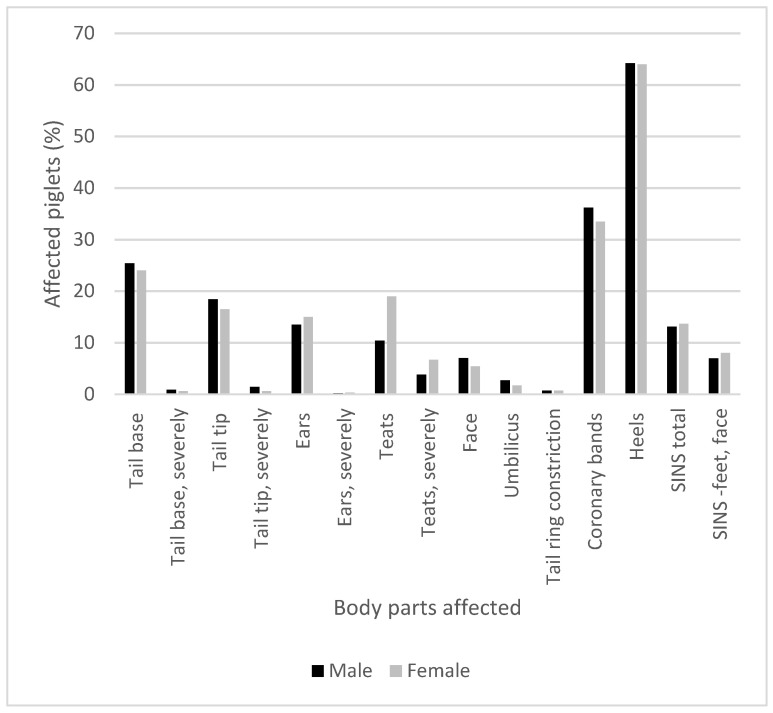
Gender differences in signs of inflammation/necrosis at different body parts in 5958 Dutch suckling piglets.

The sex of the piglets had little influence on the severity of the inflammation, except in the teats. Inflammation of the teats, both general and severe, was significantly more common in female piglets, as expected (Figure 5). In males, the tail tip, navel and face were slightly more affected than in females.

### 3.6. Correlation Between Clinical Signs of SINS in Piglets and Observation in the Sow

The herds with the 20% lowest and 20% highest SINS scores differed significantly in the status and observations made on the sows, such as body temperature, prevalence of coprostasis and average water intake (Table 5). However, uterine discharge and parity did not differ between the two groups. Piglets from affected sows not only showed a higher susceptibility to develop signs of SINS, but also had a significantly higher pre-weaning mortality rate (Table 5). Only 1% of the sows in the low SINS farms had coprostasis compared to 65% of the sows in the high SINS farms. Coprostasis was correlated with water intake, as the sows in the high SINS herds had a 20% lower average water intake than the sows in the low SINS herds.

## 4. Discussion

### 4.1. Scoring for SINS

SINS has been described as a syndrome in pigs characterised by multiple inflammatory responses, sometimes progressing to necrotic lesions, for example on the tail tip and coronary bands [22]. These lesions must be considered a serious welfare concern and require a more detailed analysis of the underlying causes in order to identify and implement corrective measures. In addition to previous studies, the aim of the current research was to re-confirm the suitability of scoring piglets for signs of SINS during their first week of life, when they are routinely handled, and to consider the impact of sow-related observations on the prevalence of SINS scores in piglets.

Scoring piglets for SINS is a non-invasive method and can be easily implemented at farm level by trained personnel as a diagnostic measure, as confirmed in the current study. The scoring system follows a predetermined matrix that converts clinical observations into binary numerical data, which are then added to metric scores, allowing simple and effective documentation of individual observations for individual animals, litters and ultimately at the herd and population levels.

Clinical signs of SINS were found in 84% of the piglets in the herds randomly selected from the client list of a veterinary practice in the Netherlands. Data analysis showed that even on the farm with the lowest incidence of SINS symptoms, 41% of the piglets had one or more visible signs of inflammation at the known predilection sites. In the top 20% of farms with the highest SINS burden, the tail base, tail tip, ears and teats were affected in 67%, 28.7%, 32.4% and 24.9% of piglets, respectively. Summarising the findings on the lower legs, such as swelling and inflammatory reactions on the coronary bands and heels, more than 70% of the piglets on 80% of the farms were not free from clinical signs of SINS.

These findings are consistent with previous reports. For example, in the study by Reiner et al. [24], 94.8, 87.5 and 40.8% of suckling piglets, weaners and finishers, respectively, were affected by SINS, without considering claw lesions, as it could not be excluded that such lesions originated from RBMs, such as the flooring system. The findings for tails, ears and teats and the higher frequency of positive SINS scores on heels and coronary bands were also confirmed in the study by Leite et al. [27] and a study from France [28]. Even in the older literature [34], tail lesions were found in 50% of litters and up to 75% of piglets with undocked tails.

### 4.2. Clinical Scores for SINS Under Field Conditions

Since the first description of SINS, the scoring system has been slightly modified by different research groups [8,26,27,28]. Despite some variations in the individual scoring matrix, the clinical signs observed are very similar and therefore data from different studies can be compared. The present study also supports the conclusion that such a scoring matrix fits its purpose, one of the key requirements for its implementation in an animal-based health and welfare assessment. However, for a full interpretation of the SINS scoring results, additional resource-based measures typical for the visited farms should be recorded. Such data should indicate the state of the farm facilities (including flooring and feeding systems, feed quality controls and overall hygiene and health management). These factors influence the prevalence of SINS-positive piglets and, more importantly, provide initial guidance for the implementation of targeted intervention strategies. However, SINS scores remain a ready-to-use and important point-of-care indicator and a valuable communication tool for farmers, nutritionists and veterinarians to improve animal welfare and avoid economic losses.

### 4.3. Environmental Factors Associated with High SINS Scores

In the data set of the current study, no specific records of the housing conditions and overall management of the farrowing unit were documented. However, due to the high prevalence of claw and heel lesions, the available data were re-analysed to test the hypothesis that the flooring in the farrowing unit was a potential driver of SINS scores. Indeed, one of the obvious and significant differences between farms was the percentage of animals with lower leg lesions (SINS+LL) and the percentage of piglets without lower leg lesions (SINS−LL). These differences persisted over time and could be considered an indicator of poor environmental conditions (RBM), such as inappropriate flooring in farrowing crates. However, the data also show that approximately 20% of the piglets had inflammatory lesions on the heels and coronary bands as early as day 1, suggesting that such inflammatory responses must have been initiated during gestation and should therefore be considered as sow-related factors contributing to the prevalence of SINS in neonatal piglets [8].

### 4.4. Sex Differences

As in previous studies [24,26], the current results did not reveal any major differences between female and male piglets, except for the prevalence of teat lesions, which was significantly (*p* < 0.05) more severe in females. Based on the hypothetical pathogenesis of SINS, mycotoxins such as zearalenone could play a role in the induction of inflammatory reactions [22]. Zearalenone is a mycotoxin commonly found in feed materials such as maize and soya beans and is known to have oestrogenic activity in all species tested. Pigs are among the most sensitive species as they convert zearalenone to its hydroxy metabolite alpha-zearalenol (α-ZEL), which has significantly higher oestrogenic activity [35]. Alpha-zearalenol can cross the placental barrier and is excreted in the milk of lactating sows, resulting in very early exposure of piglets, even at levels without obvious clinical signs of toxin exposure in the sow. As a mixed agonist/antagonist of oestrogen receptors, zearalenone and its active metabolites also have pro-inflammatory properties [36]. The greater sensitivity of female piglets to SINS suggests a possible involvement of mycotoxins.

### 4.5. Sow Factors Correlated with the Prevalence of SINS Symptoms in Piglets

The results of the current study showed a significant correlation of metabolic factors in sows such as elevated body temperature, water intake and clinical coprostasis (Table 5) when comparing the farms with the 20% lowest and 20% highest SINS scores. The close correlation between the occurrence of coprostasis and SINS in progeny confirms a previous study [24].

Inadequate water intake as described for the herds of the present study is certainly one of the major risk factors for the development of metabolic syndrome in farrowing sows, also referred to as Postpartum Dysgalactia Syndrome (PPDS) [37], and the clinical outcome can be exacerbated by fibre deficiency [38,39,40].

Coprostasis (constipation) and inadequate fluid intake lead to dysbacteriosis in the hindgut and an abundant release of bacterial endotoxins such as lipopolysaccharides (LPS). At the same time, the integrity of the intestinal barrier is compromised, leading to increased permeability for larger molecules such as LPS and even bacteria in severe cases, which then reach the submucosal area and the bloodstream [41]. Indeed, elevated LPS plasma levels as well as tumour necrosis factor alpha and interleukin 6 levels have been measured in sows with such metabolic syndrome and their piglets [42,43,44,45]. This metabolic syndrome in pregnant sows is often associated with intrauterine growth retardation (IGR) and stagnant fetal development [9,45,46,47]. It is likely that the increased percentage of stillbirths and pre-weaning losses observed in the SINS-positive herds in the current study are also associated with such a metabolic syndrome in sows with reduced water intake and coprostasis around parturition. Therefore, close monitoring of water intake in pregnant sows and adaptation of the diet to ensure adequate fibre intake in late pregnancy are recommended intervention strategies to prevent dysgalactia (PPDS and general metabolic syndrome in sows) and reduce the prevalence of SINS in piglets and weaners. In addition, sows in good health with intact teats, claws and skin have a significantly lower prevalence of SINS in their offspring from newborn piglets to finishers [24], due in part to better colostrum quality and milk production.

In addition to fibre and water intake, the quality of the sow’s diet may also contribute to the prevalence of SINS in piglets. This was demonstrated when typical tail-base lesions, as observed during SINS scoring of piglets, could be linked to the sow’s exposure to the mycotoxin deoxynivalenol [48].

Finally, it should be noted that environmental factors, such as heat stress, are becoming increasingly important and may have an impact on sow health. Heat stress is not only a result of global warming, but may also be related to the microclimate in farrowing crates. Heat stress easily compromises the intestinal barrier as a result of local hypoxia and, in the same syndrome, causes dysbacteriosis, endotoxemia and a generalised inflammatory response [10,11,12,13,49,50].

### 4.6. SINS Scores as an Indicator of Animal Health and Welfare

SINS scores appear to be associated with gut health, gut integrity and subsequently liver stability [24,32]. Sow factors such as metabolic syndrome in sows following insufficient water and fibre in the diet [43,44,45] indicate the need to monitor the gestation period of sows more closely, as even relatively simple measures such as optimal diet and unlimited water supply, and the absence of undesirable natural toxins such as mycotoxins, can reduce the risk of SINS in piglets. The SINS score has been introduced to assess animal welfare, focusing on the piglet as a physiologically vulnerable age group. However, the SINS scoring matrix allows the assessment of animal welfare and health beyond early life and weaning [24]. Gut and liver health and integrity and the subsequent systemic inflammatory response play an important role in the pathogenesis of SINS [22]. Given the evidence that the gut is an important immune organ [51], early life inflammatory responses as identified by the SINS scoring matrix are predictive of impaired development of a competent innate and adaptive immune system. In piglets with a positive SINS score, intestinal and hepatic defence and immunity are impaired [32], making the animals more susceptible to viral and bacterial infections common in young pigs. The easily visible signs of SINS therefore also serve as an important line of communication between farmers, nutritionists and farm veterinarians to explain the need for and nature of the corrective measures required. Paying particular attention to the early stages of life and the stresses associated with them, such as farrowing, birth hypoxia, rearing and weaning, will strengthen the immune competence of the individual animal and therefore its resistance to infectious diseases. Ultimately, this should also lead to a reduction in the need for antibiotics, as changes in the diet of sows and piglets and, where appropriate, the use of selected gut health stabilisers (ranging from toxin binders to pre- and probiotics and phytogenic compounds) are tools to improve health and well-being and reduce the incidence of infections requiring antibiotic treatment.

An obvious limitation of this field trial which was based on randomly enrolled farms within a given region, but with entirely different management and multiple genetics in the breeding sows and boars, is the quantitative assessment of a correlation between the SINS scores and the multiple factors that can influence the resilience of piglets to physiological stressors during late gestation (sow factors) and early life. Hence, additional studies are recommended under controlled conditions and a longer period (several rounds of gestation on an individual farm) to further identify the most relevant factors contributing to the cumulative SISN scores.

## 5. Conclusions

The well-documented SINS scoring matrix can be considered as a simple, non-invasive method to assess piglet health and welfare under field conditions. The SINS scoring system is designed to identify and report clinical signs of inflammation using a pre-set list of predilection sites for lesions in young piglets from their first day of life. Analysis of the data obtained allows the identification of technical factors such as poor flooring conditions in farrowing crates, which in most cases persist and increase in severity throughout the perinatal period. However, other pathophysiological conditions in sows and piglets that induce a systemic inflammatory response, including claw integrity and coronary band and heel lesions, must also be considered and will result in positive SINS scores. A major advantage of applying the SINS scoring matrix in the first week of life and correlating soring patterns with sow observations and feeding regimes is that early intervention strategies can be implemented to restore animal health and welfare and improve animal resistance to environmental stressors and infectious diseases.

## Figures and Tables

**Table 1:** Score matrix for clinical signs of SINS as applied in the current study.

Parameter	Score 0: No Lesions	Score 1: Lesions	Score 2: Severe Lesions
Tail base	No lesions	Oedema, swelling, redness, hairless	Ring constriction and necrotic lesions *
Tail tip	No lesions	Redness, bleeding	Necrosis
Ear base	No lesions	Hairless, shiny skin, erosion, exudate	Necrotic lesions
Teats	No lesions	Redness, swelling	Necrotic lesions
Face	No lesions	Swelling, oedema around eyes and snout, damaged skin, erosions, exudate	Multiple necrotic lesions
Claw coronary bands	No lesions	Redness, exudate, damaged skin	Necrotic lesions
Heels	No lesions	Bleeding	Necrotic lesions
Vulva	No lesions	Redness, swelling	Necrotic lesions

* Necrotic lesions: dry, black, avital areas indicating dead tissue; damaged skin: skin lesions, not necrotic.

**Table 2:** Distribution of SINS signs in individual farm visits *. The data show the percentage of affected piglets sorted by increasing percentage of SINS−LL affected piglets.

Visit	Herd	Tail Base	Tail Tip	Necrosis	Ears	Face	Teats	Umbilicus	Vulva	Coronary Bands	Heels	SINS−LL	SINS+LL
19	10	2	2	0	0	0	6	0	1	38	10	9	46.5
9	1	1	3	0	2	4	1	0	0	0	57	11	63.2
17	12	2	5	1	9	1	10	0	0	38	91	24	93.0
14	3	1	4	1	7	1	15	0	1	22	49	25	65.2
15	12	3	1	1	10	0	18	0	0	44	93	28	95.0
8	6	6	4	0	2	0	16	0	8	4	10	29	40.8
16	13	4	6	1	9	2	21	0	2	42	84	34	93.6
18	13	5	16	0	1	4	17	0	4	48	55	37	77.2
12	7	1	2	0	25	0	20	2	0	67	97	40	98.8
13	6	20	1	0	2	0	20	1	1	41	92	41	96.2
11	5	25	3	1	17	3	20	0	2	52	54	51	81.7
10	13	4	16	3	35	0	18	3	0	49	97	54	98.7
1	9	37	51	0	9	16	21	10	2	54	79	71	94.9
2.1	8	69	8	0	1	0	1	0	0	1	51	71	82.9
2	8	7	51	0	13	24	0	20	3	19	70	74	90.6
6	5	64	3	0	14	1	33	1	1	41	61	77	92.0
7	13	56	12	0	54	42	31	0	2	33	43	77	82.8
5	2	54	69	0	44	7	16	0	7	0	0	88	87.9
4	11	85	33	2	7	4	16	0	1	34	63	91	95.4
3	4	84	50	2	22	14	22	3	0	48	73	93	97.7

* Individual farms were visited more than once to observe the effects of climate and management. However, these data could not be included because of high variability. SINS: additive total SINS scores including SINS+LL; SINS−LL: percentage of piglets showing signs of SINS but excluding lesions at the heels and coronary bands; both data rows were calculated from the total numbers of piglets at SINS-positive farms.

**Table 3:** Signs of SINS in 20% herds with lowest and 20% of herds with highest SINS status.

	SINS-Status in Herds		
Piglets with signs at	low	high	*p*-value
Tail base (%)	1.4	67.0	<0.001
Tail Base (severe) (%)	0.2	0.9	n.s. *
Tail tip (%)	3.0	28.7	<0.001
Tail tip (severe) (%)	0.1	0.3	n.s.
Tail ring formation (%)	0.4	0.9	n.s.
Ears (%)	2.9	32.4	<0.001
Ears (severe) (%)	0.1	0.3	n.s.
Face (%)	1.2	19.9	<0.001
Teats (%)	8.2	24.9	<0.001
Teats (severe) (%)	3.6	2.3	n.s.
Umbilicus (%)	0.0	0.7	0.008
Vulva (%)	1.2	2.2	n.s.
Vulva (severe) (%)	0.0	0.2	n.s.
Coronary bands (%)	22.4	32.9	<0.001
Heels (%)	34.1	49.3	<0.001
SINS+LL (%)	56.3	89.8	<0.001
SINS−LL (%)	15.0	82.7	<0.001

* n.s. = not significant.

**Table 4:** Correlation between SINS+LL and SINS−LL within the first 7 days of life (n = 5958 scores from individual piglets).

Days of Life of Piglets a at Scoring	r SINS+LL/ SINS−LL	r SINS+LL01 (%)/ SINS−LL01 (%)	Sensitivity (%)
1	0.875	0.835	88
2	0.811	0.527	61
3	0.798	0.303	49
4	0.838	0.281	54
5	0.884	0.133	37
6	0.835	0.228	38
7	0.866	0.226	31
1–7	0.815	0.381	53

r: correlation coefficient; r SINS+LL/SINS−LL: correlation of SINS scores including heels and coronary bands (SINS+LL) with SINS scores excluding heels and coronary bands (SINS−LL); r SINS+LL01/SINS−LL01: correlation between the identification of a piglet being SINS-positive, including (SINS+LL) and excluding (SINS−LL) heels and coronary bands; sensitivity regarding r SINS+LL01/SINS−LL01.

**Table 5:** Findings in sows comparing herds with 20% lowest and the herds with 20% highest SINS scores (80% percentile).

		Herds with	Herds with	*p*
		Lowest SINS Scores	Highest SINS Scores	
Body temperature (sow)	Mean	38.81	39.04	<0.001
	SD	0.59	0.49	
	CI	38.9–38.8	39.0–39.1	
Coprostasis (sow)	Mean	0.01	0.65	<0.001
	SD	0.12	0.49	
	CI	0.01–0.02	0.46–0.85	
Average water intake	Mean	11.3	9.05	<0.001
(sow)	SD	2.50	1.59	
	CI	11.16–11.53	8.92–9.19	
Uterine discharge (sow)	Mean	0.26	0.22	n.s.
	SD	0.44	0.41	
	CI	0.23–0.29	0.18–0.25	
Parity (sow)	Mean	4.03	4.03	n.s.
	SD	2.17	2.59	
	CI	3.89–4.16	3.87–4.18	
Preweaning piglet losses	Mean	0.79	2.62	<0.001
	SD	0.87	1.55	
	CI	0.73–0.84	2.53–2.71	

SD: standard deviation; CI: 95% confidence interval; *p*: significance; n.s.; not significant.

## Data Availability

Any data used or analysed during the current study are available from the authors on reasonable request.

## Abbreviations

The following abbreviations are used in this manuscript:

ABM	Animal-based measure
CI	Confidence interval, 95%
IGR	Intrauterine growth retardation
LPS	Lipopolysaccharide
n.s.	Not significant
*p*	Significance
PPDS	Postpartum Dysgalactia Syndrome
R^2^	Coefficient of determination
RBM	Resource-based measure
SD	Standard deviation
SINS	Swine Inflammation and Necrosis Syndrome
SINS−LL	SINS, excluding lower leg lesions (coronary bands and heels)
SINS+LL	SINS, including coronary bands and heels
ZEL	Zearalenol
